# Conjunctival squamous cell carcinoma with massive apoptosis and immune cell infiltration: A case report

**DOI:** 10.3389/fsurg.2022.1004554

**Published:** 2022-10-14

**Authors:** Gang Du, Jun Qiao, Xunwen Lei, Ruiqin Han

**Affiliations:** ^1^ Ophthalmology Department, First Hospital of Lanzhou University, Lanzhou, China; ^2^ Lanzhou Ophthalmology Center Affiliated to Gansu University of Chinese Medicine, Lanzhou Huaxia Eye Hospital, Lanzhou, China; ^3^ State Key Laboratory of Medical Molecular Biology, Department of Biochemistry and Molecular Biology, Institute of Basic Medical Sciences, Chinese Academy of Medical Sciences and Peking Union Medical College, Beijing, China

**Keywords:** conjunctival squamous cell carcinoma, SCC, apoptosis, immune cell infiltration, case—based content filtering algorithm

## Abstract

This case reports a rare case of conjunctival squamous cell carcinoma in China. The elderly (86-year-old) female patient was diagnosed and treated effectively after three times of diagnosis. During this period, she was misdiagnosed and ineffective treatment for many times. Therefore, we propose to make an integrated diagnosis based on histopathological diagnosis, combined with a variety of diagnostic methods including MRI and CDFI, supplemented by updated multiple immunohistochemically techniques, so as to achieve the purpose of accurate diagnosis.

## Introduction

Conjunctival squamous cell carcinoma (SCC) is the end stage of a series of diseases called ocular surface squamous neoplasia (OSSN), which is a malignant eye disease, which can lead to vision loss and death in severe cases (1, 2). The main risk factors for this disease are exposure to outdoor solar ultraviolet radiation, HIV/AIDS, human papillomavirus and allergic conjunctivitis (3–5). The incidence rate of conjunctival squamous cell carcinoma is the highest in Africa. About 1.3 of every 100 thousand people suffer from conjunctival squamous cell carcinoma each year (6). In contrast, the incidence rate in other areas is about 0.1 per 100 thousand populations per year, 10 times lower than that in other regions (7). The incidence rate of SCC decreased by 49% with the increase of latitude 10°. The isolated incidence rate of Denmark (55–57° north) was 0.02 per 100,000 males per year and 0.008 per 100,000 females, which is closely related to other studies in the northern high latitude area (8). The disease has many manifestations. Red eyes, photophobia, irritation, foreign body sensation and white, painless and progressive growth on the surface of the eyes are common symptoms (9). Most lesions occur in blepharoplasty, especially on the nasal side (10). Histopathology is the gold standard of diagnosis: pathologists will see histopathological changes between normal and abnormal tissues1. However, histopathology is not without challenges. Resection requires surgical intervention, and the explanation is subjective. Different pathologists have different explanations. The main treatment of OSSN is complete resection, which may lead to recurrence in about 5% of patients, while incomplete resection may lead to recurrence in up to 56% of patients (10, 11). Since complete resection is often difficult to achieve, many adjuvant therapies have been developed to reduce the risk of recurrence. These therapies include cryotherapy during resection and radiotherapy or local chemotherapy after resection (12–18).

Unfortunately, most cases are diagnosed according to clinical impression. About half of the lesions were not resected for histopathological examination. This may be related to the increasing trend of major local drugs in the treatment of these lesions. However, the clinical impression is unreliable, especially in areas with poor medical conditions, because both benign and malignant lesions have overlapping characteristics. In the absence of tissue diagnosis, the use of potentially dangerous local drugs (such as cytotoxic drugs) can be counterproductive.

Therefore, based on histopathological diagnosis, the combination of multiple diagnostic methods is very important for the accurate diagnosis of SCC. SCC is a kind of rare cancer in China, we hereby present the rare case of an old woman, combined diagnosed by conjunctival squamous cell carcinoma using kinds of different diagnosis methods, and investigation the apoptosis and immune cell infiltration by the updated mIHC. It provides a new idea for the diagnosis and treatment of SCC.

## Case presentation

### 1st diagnosis and treatment

The patient, female, 86 years old, felt that her left eye was red and secretions increased in May 2020. She went to the local clinic and was given drug treatment (the specific medication is unknown). But the curative effect was poor. She went to the local hospital seven months later and was diagnosed as “left eye tumor”. Then, she was recommended to go to the superior hospital for diagnosis and treatment.

### 2nd diagnosis and treatment

On 27 January 2021, the patient went to the Lanzhou University Second Hospital and was diagnosed as “left eyelid tumor”. On 1 February 2021, histopathological diagnosis was: (left eyelid conjunctiva) squamous epithelial papilloma, mild to moderate atypical hyperplasia of epithelium, accompanied by suppurative inflammation. Therefore, “left eye conjunctival cryosurgery” was performed under local anesthesia on 22 February 2021 and 16 March 2021.

### 3rd diagnosis and treatment

The patient felt that the tumor in his left eye increased rapidly, accompanied by obvious swelling and pain, loss of vision and difficulty in opening her eyes. She was treated in the first people’s hospital of Lanzhou city on 7 April 2021. The diagnosis was: space occupying in the left eye orbit, and surgical treatment was recommended.

The patient was admitted to Lanzhou Huaxia ophthalmic hospital for surgical treatment on 20 April 2021.

**Figure 1:**
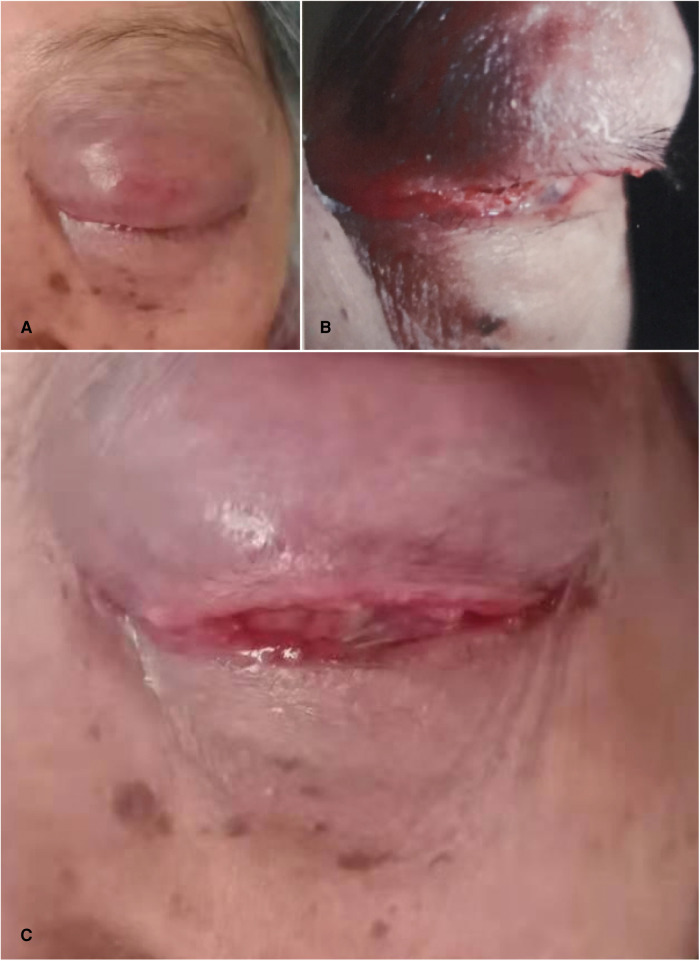
Ocular pathological features. (**A**) the patient’s left upper eyelid was red and swollen, the skin lines disappeared, and it was difficult to open his eyes. (**B**) the patient's upper eyelid can reach a size of about 30 mm × 20 mm mass with unclear boundary, hard quality and poor activity. (**C**) the patient had bulbar conjunctival congestion, edema and corneal conjunctivalization.

**Eye examination:** The upper eyelid of the left eye is red and swollen, the dermatoglyphs disappear, and it is difficult to open the eyes. The upper eyelid can touch a size of about 30 mm  ×  20 mm mass with unclear boundary, hard texture, poor activity, obvious tenderness, bulbar conjunctival congestion and edema, corneal conjunctivalization, and other structures cannot be seen clearly; the international standard visual acuity chart was used to check the visual acuity: the naked visual acuity was 0.2 in the right eye and no light perception in the left eye; the intraocular pressure was measured by non-contact tonometer: 11.5 mmHg (1 mmHg = 0.133 kpa) in the right eye and 46.5 mmHg (1 mmHg = 0.133 kpa) in the left eye (Figures 1A–C).

Ocular ultrasonography: the upper part of the left eyeball is about 30 mm × 19 mm heterogeneous hypoechoic mass with unclear boundary, pressed close to the eyeball wall and sunken into the ball.

**Color Doppler flow imaging (CDFI):** point and strip blood flow signals can be seen in eyeball.

**Figure 2:**
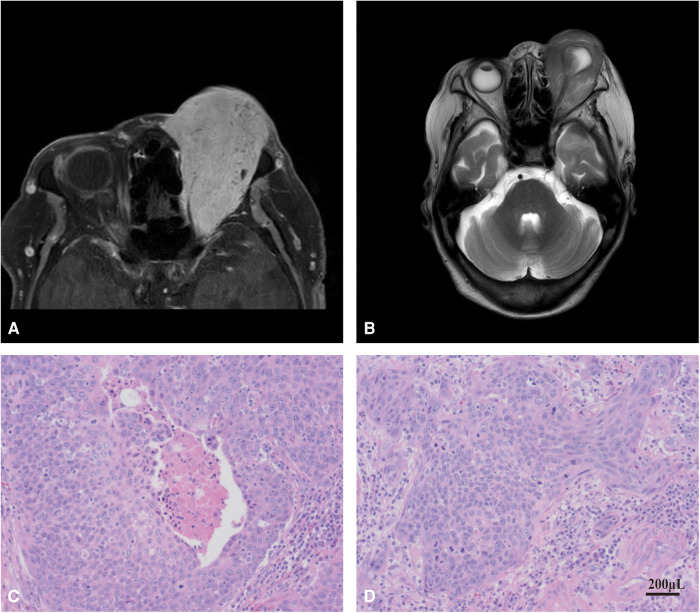
MRI examination and histopathological verification. (**A**) MRI examination of the patient's orbit. (**B**) enhanced MRI of the patient's orbit. (**C,D**), histopathological verification of tumor tissue in SCC patient.

**Enhanced MRI examination of orbit and orbit:** irregular soft tissue signal shadow can be seen in the left orbital diaphragm, inside and outside the muscle cone and lacrimal gland area, which is filled in the orbit as frozen and extends backward into the optic nerve canal. T1W1 shows equal signal, T2W1 and DW1 show slightly high signal, the internal signal is uneven, and the space occupying effect is obvious. The left eyeball is compressed, deformed and moved forward, the eyeball wall collapses, and the left optic nerve is compressed, the external rectus muscle of lacrimal gland was not clearly displayed. The left orbit and optic canal are enlarged, and no abnormality is found in the bone. The enhancement scan was performed immediately after 15 ml gadolinium diamine contrast agent was injected through elbow vein. It showed that the signal shadow of soft tissue in left diaphragm, inside and outside muscle cone and lacrimal gland area was significantly enhanced, and there was spotted low signal shadow inside, and there were no abnormal enhancement changes in the rest (Figures 2A,B).

**Exclude cancer metastasis diagnosis:** In order to exclude metastatic cancer, the patient underwent systemic examination, and no abnormality was found in gynecological examination; No space occupying lesions were found in brain MRI, chest x-ray examination, neck and abdominal ultrasonography (liver, gallbladder, pancreas, spleen and double kidneys); No obvious abnormality was found in liver and kidney function and blood routine. Metastatic cancer and systemic lesions can be excluded.

**Left eye orbital tumor resection:** Due to the patient's old age and the request of her family to retain the patient's eyeball, the “left eye orbital tumor resection” was performed under general anesthesia on 22 April 2021. The postoperative histopathological diagnosis was: (left eye) malignant tumor, consistent with squamous cell carcinoma, with extensive infiltration of cancer tissue into stroma and multifocal necrosis (Figures 2C,D).

**Figure 3:**
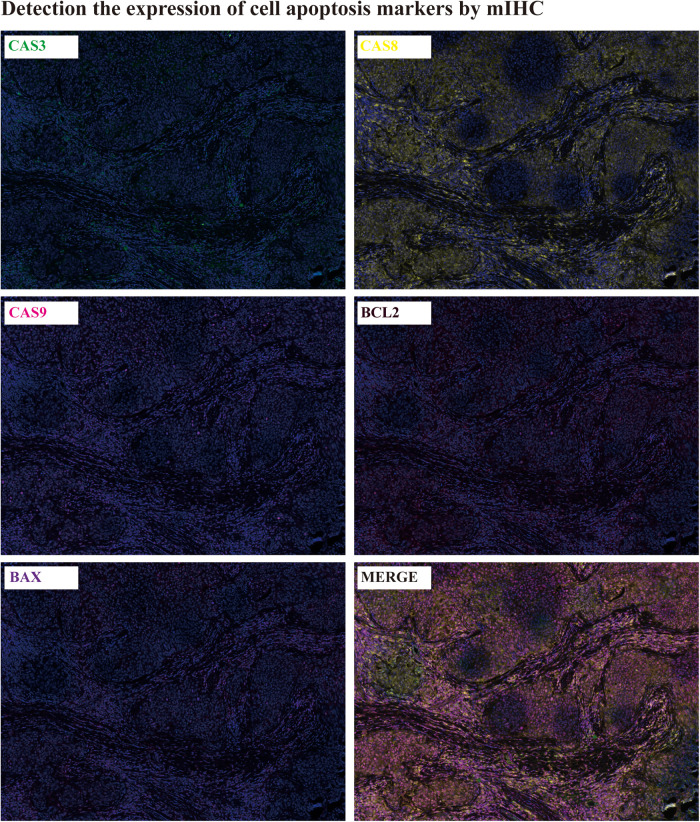
Detection the cell apoptosis by mIHC.

**Apoptosis detection:** The cell apoptosis in patient tumor tissues was detected by mIHC(mutiplex immunohistochemistry). The expression of CASPASE3,8,9 and BCL2, BAX were determined by mIHC. It was showed that all of the apoptosis markers were extensively expressed in the tumor tissues, which infer to there was ubiquitous apoptosis in tumor tissues (Figure 3).

**Figure 4:**
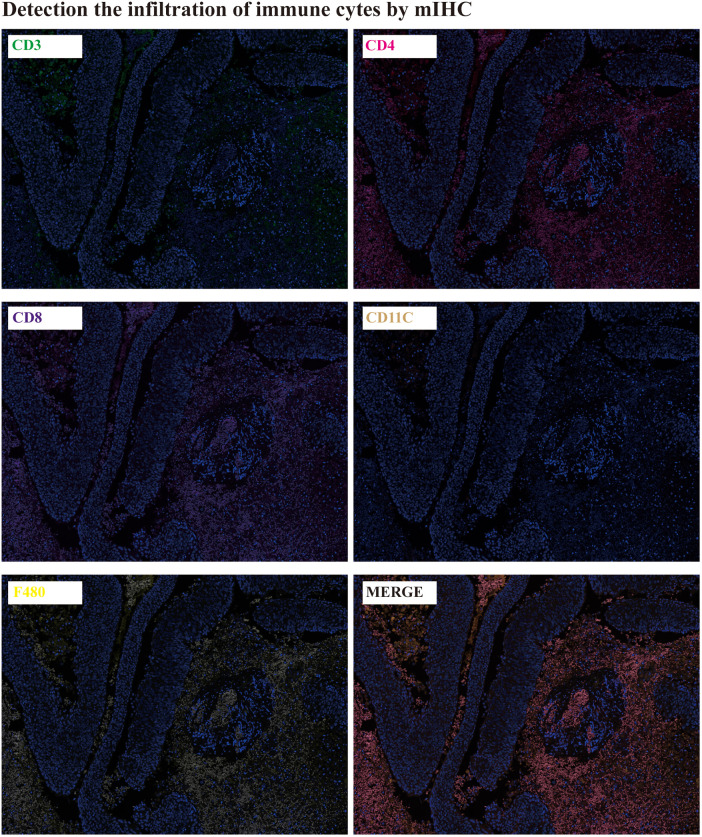
Detection the immune cells infiltration by mIHC.

**The immune cells infiltration detection:** The immune cells infiltration in tumor tissues were detected by mIHC. CD3 is the total T lymphocyte marker, CD4 is the helper T lymphocyte marker, CD8 is the cytotoxic T lymphocyte marker, CD11C is the natural killer cell, and F480 is the macrophage marker. It was showed that there were lots of CD3, CD4, CD8 and F480 expression in tumor tissues, except for CD11C (Figure 4).

## Discussion

SCC of the conjunctiva is an uncommon lesion in China, it often occurs in the limbus of the eyelid fissure area, the junction of the eyelid margin skin and conjunctiva, or the lacrimal caruncle of the inner canthus, and rarely in the non-exposed area of the conjunctiva. Some tumors look like pterygium. Most of the tumors were glial and epithelial hyperkeratosis. The tumor grows slowly, but can infiltrate into deep tissue and rarely metastasize.

It is difficult to distinguish between dysplasia, carcinoma *in situ* (CIS) and squamous cell carcinoma only from a clinical point of view (19). The overall clinical accuracy of 288 patients with OSSN (including 62 cases of squamous cell carcinoma) by Lee and Hirst was 33%, and only 30% in the squamous cell carcinoma group. The macroscopic morphology of squamous cell carcinoma is described as gelatinous, velvet or papillary, leukoplakia, nodular and diffuse (20–22). Because of its morphological diversity, it is a challenge for accurate diagnosis. We report a case in which the upper eyelid of the patient's left eye was red and swollen, the skin lines disappeared, and it was difficult to open his eyes. The upper eyelid could reach a size of about 30 mm × 20 mm mass with unclear boundary, hard texture, poor activity, obvious tenderness, bulbar conjunctival congestion and edema, corneal conjunctivization, and other structures cannot be seen clearly.

It was found that the tumors mainly occurred in elderly men (77%), and most of the lesions involved the limbal (81%). All patients were white or equatorial Africans, and other races were less affected by the disease. Conjunctival squamous cell carcinoma is more common in elderly male patients. It often occurs in bulbar conjunctival tissue near the conjunctival edge of the corner of the palpebral fissure (23, 24). But, studies show that the prevalence of disease in the tropics and subtropics is younger, and the incidence rate of young people is higher than that of the elderly (19). In addition to, we found that the disease is often accompanied by large-area necrosis and cell apoptosis and massive infiltration of immune cells, this was consent to the other cancer study (25, 26).

Due to the morphological and pathological diversity of SCC, the patients in our reported cases have experienced many times of diagnosis and treatment. Although they have finally obtained more effective treatment, it virtually increases the economic burden and physical and mental pain of patients, and prolongs the treatment cycle of the disease. In order to avoid similar situations, in addition to using the gold standard of histopathology, we integrate various existing diagnostic techniques, such as MRI, CDFI and so on.

## Conclusion

SCC is a race specific or region-specific eye disease. It mostly occurs in tropical and subtropical areas such as equatorial Africa or Australia. The incidence of SCC is low in China. Due to its various clinical manifestations, it is difficult to accurately diagnose the disease. Histopathological diagnosis is the gold standard for the diagnosis of the disease. Our clinical research found that histopathological diagnosis is the main diagnosis, and the combination of multiple diagnostic methods may achieve the purpose of faster and more accurate diagnosis. At the same time, we found that the disease is often accompanied by large-area necrosis and cell apoptosis and massive infiltration of immune cells, which provides a new idea for its diagnosis and treatment.

## Data Availability

The original contributions presented in the study are included in the article/Supplementary Material, further inquiries can be directed to the corresponding author/s.

## References

[1] Gichuhi S Sagoo MS. Squamous cell carcinoma of the conjunctiva. Community Eye Health. (2016) 29:52–3. PMID: ; PMCID: 28289320 PMC5340104

[2] Serna-Ojeda JC Hernandez-Orgaz J Olvera-Morales O. [Squamous cell carcinoma of the Conjunctiva]. Rev Fac Cien Med Univ Nac Cordoba. (2017) 74:402–4. 10.31053/1853.0605.v74.n4.16922 29902151

[3] Merz LE Afriyie O Jiagge E Adjei E Foltin SK Ludwig ML Clinical characteristics, HIV status, and molecular biomarkers in squamous cell carcinoma of the conjunctiva in Ghana. Health Sci Rep. (2019) 2:e108. 10.1002/hsr2.108 30809594 PMC6375545

[4] Salceanu SO Constantin C Cijevschi I Ursu RG Grigorovici M Iancu LS. Human papillomavirus 52 positive squamous cell carcinoma of the conjunctiva. Indian J Ophthalmol. (2015) 63:166–9. 10.4103/0301-4738.154406 25827551 PMC4399129

[5] Starita N Annunziata C Waddell KM Buonaguro L Buonaguro FM Tornesello ML. Identification of human herpesvirus 8 sequences in Conjunctiva intraepithelial neoplasia and squamous cell carcinoma of Ugandan patients. Biomed Res Int. (2015) 2015:801353. 10.1155/2015/801353 26509162 PMC4609772

[6] Hammerl L Ferlay J Borok M Carrilho C Parkin DM. The burden of squamous cell carcinoma of the conjunctiva in Africa. Cancer Epidemiol. (2019) 61:150–3. 10.1016/j.canep.2019.06.007 31255960

[7] Emmanuel B Ruder E Lin SW Abnet C Hollenbeck A Mbulaiteye S. Incidence of squamous-cell carcinoma of the conjunctiva and other eye cancers in the NIH-AARP Diet and Health Study. Ecancermedicalscience. (2012) 6:254. 10.3332/ecancer.2012.254 22654961 PMC3357182

[8] Fagerberg PS Ramberg IMS Toft PB. Combining brachytherapy and cryotherapy as adjuvant therapy for squamous cell carcinoma of the Conjunctiva: literature review and case reports. Ocul Oncol Pathol. (2021) 7:77–84. 10.1159/000512029 33981690 PMC8077545

[9] Waddell KM Downing RG Lucas SB Newton R. Corneo-conjunctival carcinoma in Uganda. Eye (Lond). (2006) 20:893–9. 10.1038/sj.eye.6702043 16456599

[10] Tunc M Char DH Crawford B Miller T. Intraepithelial and invasive squamous cell carcinoma of the conjunctiva: analysis of 60 cases. Br J Ophthalmol. (1999) 83:98–103. 10.1136/bjo.83.1.98 10209445 PMC1722787

[11] Gichuhi S Sagoo MS Weiss HA Burton MJ. Epidemiology of ocular surface squamous neoplasia in Africa. Trop Med Int Health. (2013) 18:1424–43. 10.1111/tmi.12203 24237784 PMC4440345

[12] Lecuona K Stannard C Hart G Rice J Cook C Wetter J The treatment of carcinoma in situ and squamous cell carcinoma of the conjunctiva with fractionated strontium-90 radiation in a population with a high prevalence of HIV. Br J Ophthalmol. (2015) 99:1158–61. 10.1136/bjophthalmol-2014-306327 25784215

[13] Al-Barrag A Al-Shaer M Al-Matary N Al-Hamdani M. 5-Fluorouracil For the treatment of intraepithelial neoplasia and squamous cell carcinoma of the conjunctiva, and cornea. Clin Ophthalmol. (2010) 4:801–8. 10.2147/OPTH.S9709 20689797 PMC2915867

[14] Chaugule SS Park J Finger PT. Topical chemotherapy for giant ocular surface squamous neoplasia of the conjunctiva and cornea: is surgery necessary? Indian J Ophthalmol. (2018) 66:55–60. 10.4103/ijo.IJO_590_17 29283124 PMC5778583

[15] Kalamkar C Radke N Mukherjee A Radke S. Topical mitomycin-C chemotherapy in ocular surface squamous neoplasia. J Clin Diagn Res. (2016) 10:NJ01. 10.1111/crj.12367 PMC5071982 27790482

[16] Pe'er J. Ocular surface squamous neoplasia: evidence for topical chemotherapy. Int Ophthalmol Clin. (2015) 55:9–21. 10.1097/IIO.0000000000000050 25436490

[17] Rudkin AK Dempster L Muecke JS. Management of diffuse ocular surface squamous neoplasia: efficacy and complications of topical chemotherapy. Clin Exp Ophthalmol. (2015) 43:20–5. 10.1111/ceo.12377 24995542

[18] Nanji AA Sayyad FE Karp CL. Topical chemotherapy for ocular surface squamous neoplasia. Curr Opin Ophthalmol. (2013) 24:336–42. 10.1097/ICU.0b013e3283622a13 23680759

[19] Lee GA Hirst LW. Retrospective study of ocular surface squamous neoplasia. Aust N Z J Ophthalmol. (1997) 25:269–76. 10.1111/j.1442-9071.1997.tb01514.x 9395829

[20] Iliff WJ Marback R Green WR. Invasive squamous cell carcinoma of the conjunctiva. Arch Ophthalmol. (1975) 93:119–22. 10.1001/archopht.1975.01010020125005 1115670

[21] Bullock JD Albert DM Richman SJ. Squamous cell carcinoma of the conjunctiva. Ohio State Med J. (1974) 70:502–3. PMID: 4845243

[22] Blodi FC. Squamous cell carcinoma of the conjunctiva. Doc Ophthalmol. (1973) 34:93–108. 10.1007/BF00151799 4706112

[23] McKelvie PA Daniell M McNab A Loughnan M Santamaria JD. Squamous cell carcinoma of the conjunctiva: a series of 26 cases. Br J Ophthalmol. (2002) 86:168–73. 10.1136/bjo.86.2.168 11815342 PMC1770993

[24] Cervantes G Rodriguez AA Jr. Leal AG. Squamous cell carcinoma of the conjunctiva: clinicopathological features in 287 cases. Can J Ophthalmol. (2002) 37:14–9; discussion 19–20. 10.1016/S0008-4182(02)80093-X 11865953

[25] Xie J Chen L Tang Q Wei W Cao Y Wu C A necroptosis-related prognostic model of uveal melanoma was constructed by single-cell sequencing analysis and weighted co-expression network analysis based on public databases. Front Immunol. (2022) 13:847624. 10.3389/fimmu.2022.847624 35242144 PMC8886618

[26] Xie J Chen L Cao Y Wu D Xiong W Zhang K Single-cell sequencing analysis and weighted co-expression network analysis based on public databases identified that TNC is a novel biomarker for keloid. Front Immunol. (2021) 12:783907. 10.3389/fimmu.2021.783907 35003102 PMC8728089

